# Supplementary material for: Altered N6-Methyladenosine Modification Patterns and Transcript Profiles Contributes to Cognitive Dysfunction in High-Fat Induced Diabetic Mice
Source: Int J Mol Sci. 2024 Feb 6;25(4):1990. doi: 10.3390/ijms25041990 (PMC10889299; doi:10.3390/ijms25041990)
Supplement: Supplementary file 1 [file ijms-25-01990-s001.zip › ijms-2802135-supplementary.pdf]

Table S1. Primers used in this study

Gene	Primer type	Primer Sequence （5'-3'）
FTO	Forward	GACACTTGGCTTCCTTACCTG
FTO	Reward	CTCACCACGTCCCGAAACAA
METTL3	Forward	CTGGGCACTTGGATTTAAGGAA
METTL3	Reward	TGAGAGGTGGTGTAGCAACTT
METTL14	Forward	CTCCAAACTCAAAACGGAAGTGT
METTL14	Reward	ATGGGGATTTAAGCTCTGCGT
